# Real-Time Fluorescence-Based Method for Dynamic Quantification of Droplet Network Assembly

**DOI:** 10.1021/acsomega.5c02156

**Published:** 2025-06-02

**Authors:** Alessia Faggian, Federica Casiraghi, Martin M. Hanczyc

**Affiliations:** † Laboratory for Artificial Biology, Department of Cellular, Computational and Integrative Biology, University of Trento, Via Sommarive, 9, Povo 38123, Italy; ‡ Chemical and Biological Engineering, University of New Mexico, Albuquerque, New Mexico 87106, United States

## Abstract

This study introduces a direct fluorescence-based molecular beacon method to monitor droplet assembly in real-time, enhancing the precision of synthetic biology and mesoscale material applications. Unlike traditional imaging techniques, such as Pearson correlation from microscopic images, the direct method allows continuous quantification of dynamic droplet interactions with high sensitivity. This system utilizes single-stranded DNA (ssDNA) beacons that fluoresce upon binding with complementary ssDNA sequences in adjacent droplets, enabling the specific detection of assembly events. The ability to manipulate assembly accurately expands potential applications including the design of programmable cellular mimics, biosensors for environmental monitoring, and smart drug delivery systems that respond to specific cellular cues.

## Graphical abstract

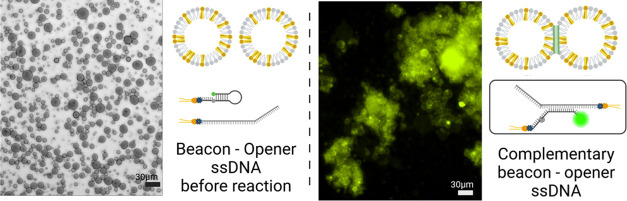

## Introduction

Controlled assembly of biomolecular complexes plays a crucial role in biology at all levels of organization, from the inner mechanics of a cell to the global response of an organism to a stimulus.^1–3^ Recently, much attention has been given to the role of liquid–liquid phase-separated droplets within a cell based on the assembly of RNA–protein complexes, signifying that such interactions may play critical roles in the functionality of a cell.^4^ In synthetic biology,^5,6^ the controlled and programmable organization of droplet networks serves as a powerful model for cellular processes, with applications in diagnostics, drug delivery, and metabolic engineering. Understanding and quantifying these dynamic assembly processes in real-time are critical for advancing responsive systems design that mimics biological function. Traditional methods for studying droplet assembly rely on imaging-based techniques that quantify fluorescence overlap between different components, such as the Pearson correlation coefficient.^6^ However, these methods often fall short when it comes to distinguishing between closely spaced droplets or resolving specific molecular interactions within droplet networks. To address these limitations, we introduce a fluorescence-based molecular beacon method that allows for real-time, quantitative analysis of droplet network assembly with high sensitivity.^7–9^ This method not only offers a more straightforward and flexible approach compared to traditional fluorescence correlation techniques but also provides direct measurements of physical connections among droplets. Through this fluorescence-based molecular beacon approach, we can quantitatively follow the evolution of droplet assemblies and gain deeper insights into the kinetics of droplet interactions.

## Results and Discussion

Distinct emulsified droplet (ED) populations were decorated with the beacon, with a complementary opener (see Figure 1), or with noncomplementary ssDNA as a control. Binary mixes were made and observed by microscopy. We observe a distinct difference between assembled and unassembled droplets, which differ only by their complementary or noncomplementary ssDNA information. Unassembled droplets appear as a lawn of distributed EDs, and assemblies on the other hand are visible as clusters (Figure 2). The green signal is observed when the beacon is unquenched, indicating that the beacon opening is specific to its designated complementary opener sequence. In the negative control, the beacon population is mixed with a population labeled as noncomplementary ssDNA. Nonassembled EDs with noncomplementary DNA exhibit a faint and pale green color, representing the thermodynamic opening of the beacon as a background signal (Figure 2A).

**Figure 1:**
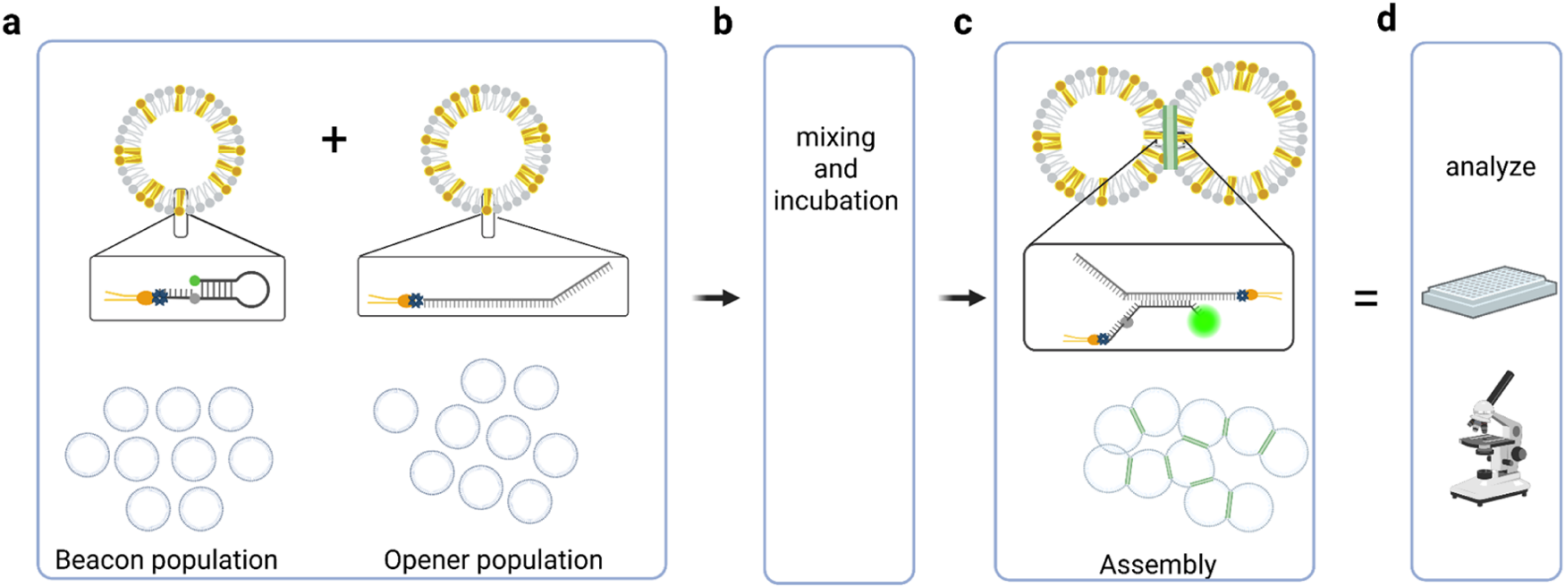
Schematic illustration of the workflow. (a) Two populations of droplets are separately functionalized with either molecular beacon DNA or a complementary (opener) ssDNA linked to a modified lipid by a biotin–streptavidin–biotin bridge (dark-yellow lipid). (b) Two populations are mixed and incubated under conditions that permit hybridization and interaction between complementary DNA strands. (c) When the two populations are mixed, the molecular beacon is open and the fluorophore unquenched, resulting in an assembly with a clear fluorescence green signal. (d) Outcome analyzed via microscopy and/or a plate reader.

**Figure 2:**
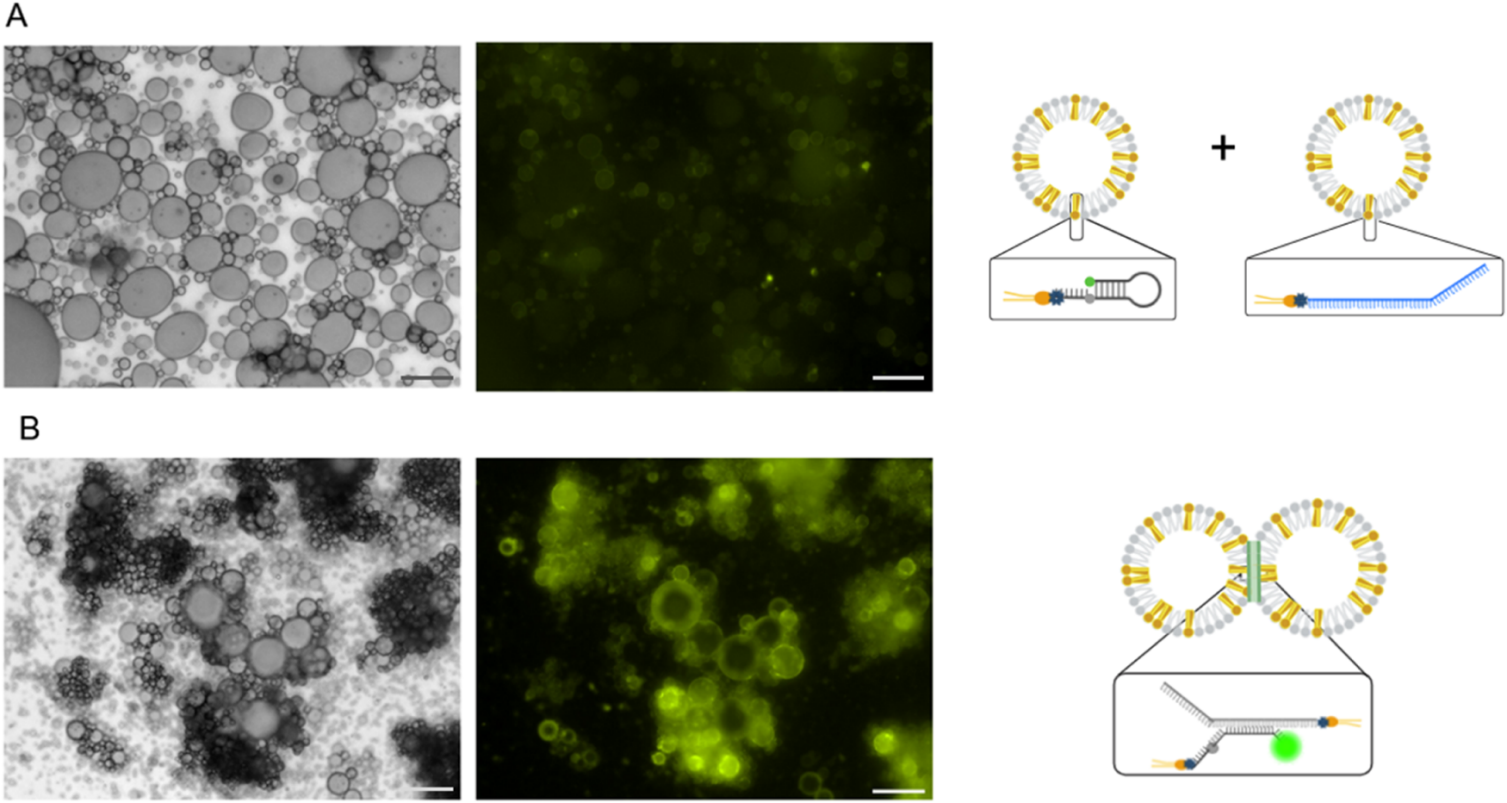
Microscopic view of the beacon activity. Bright-field (left) and fluorescence (right) of (A) negative control (droplets functionalized with ssDNA beacon + droplets functionalized with ssDNA random sequence (blue)) and (B) assembled droplets (droplets functionalized with ssDNA beacon + droplets functionalized with ssDNA opener); the fluorescence signal indicates the binding of DNA beacon to its opener. Schematic cartoons (right) illustrate the respective molecular configurations under each condition. Scale bar: 50 μm.

To demonstrate the sensitivity of this technique, varying ratios of EDs with beacon and EDs with the opener were tested for the fluorescence signal over time; see Figure 3. The data clearly distinguish the insufficient assembly observed with the 1/25x beacon +2x opener condition from the highly efficient and rapid assembly achieved with the 1/5x beacon +4x opener condition. At a low concentration of 1/25x beacon, even 2x opener is insufficient to generate a detectable signal, likely due to a threshold effect where the beacon concentration is too low for efficient interaction. Conversely, 1/10x beacon is sufficient to support assembly across a range of opener concentrations (2x–4x), with fluorescence intensities increasing progressively and reaching a plateau at 4x opener, suggesting maximal efficiency under these conditions. At the highest tested concentration, 1/5x beacon, the assembly remains highly efficient even at 4x opener, with fluorescence intensity still increasing beyond 400 min, indicating an extended kinetic response and suggesting that the system remains sensitive to further opener-induced assembly over time. This graded response underscores the method’s robustness for quantifying assembly dynamics across a range of conditions.

**Figure 3:**
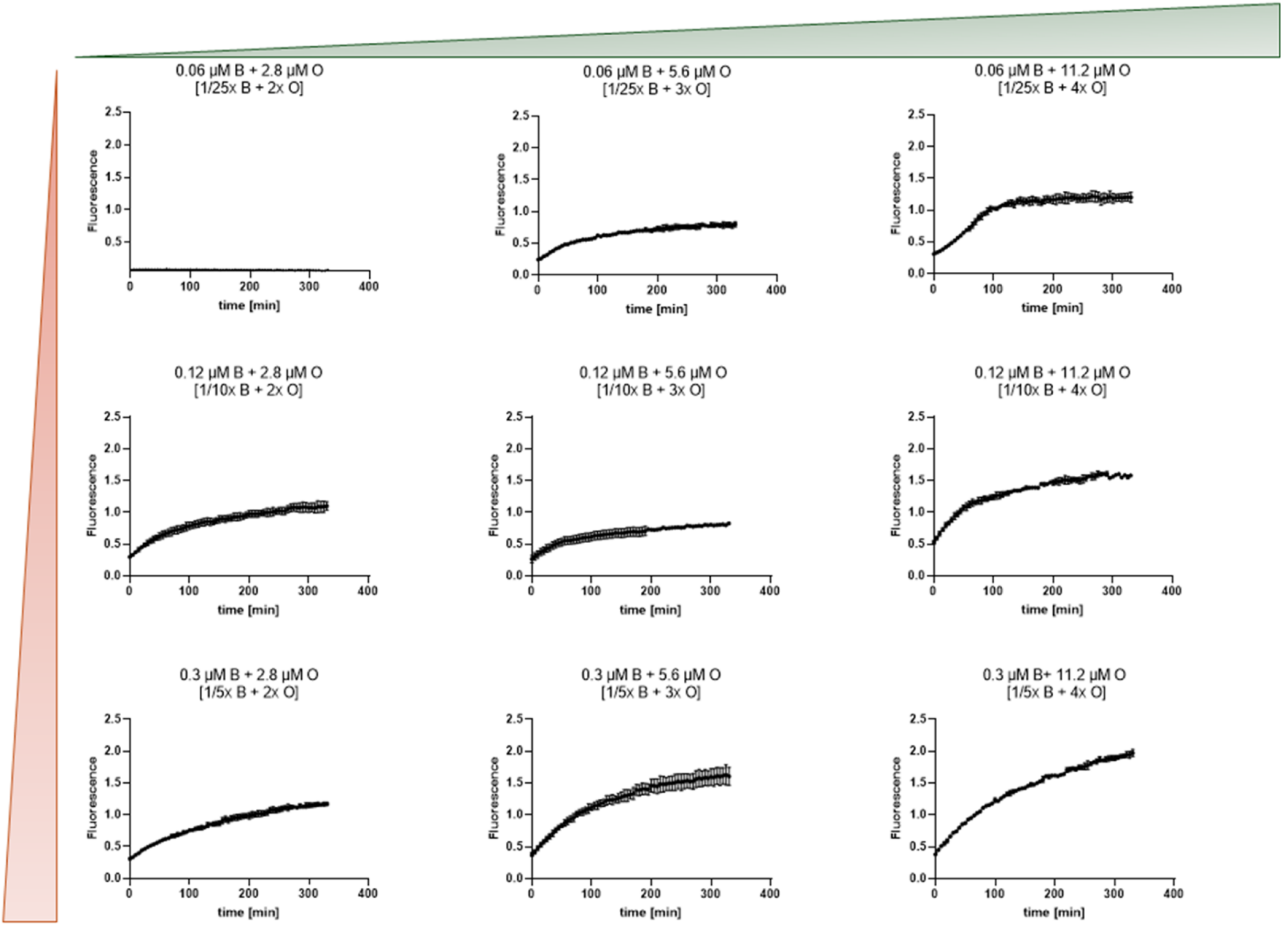
Fluorescence intensity curves plotted against time for various experimental conditions involving combinations of a beacon (B) and opener (O). Each row of plots represents a different concentration of B, while each column shows increasing concentrations of O. The green triangle at the top represents an increasing concentration of the opener, the red triangle to the side represents an increasing concentration of the beacon. Fluorescence values (*y*-axis) are a direct reading from the instrument; each experiment was performed with four technical replicates (quadruplets), and four independent experiments were conducted, carried out on separate days, using distinct batches of droplets. Each point represents the average of a quadruplet. In [brackets] “*x*” fold of the standard amount used in the pixel-based colocalization method provided for comparison of the two methods.

In our previous publications, we used complementary linear ssDNAs to govern the higher-order assembly of droplets^10^ with a pixel-based colocalization method to calculate the assembly where the fluorescence signal came from dyes associated with the lipids at the interface. In other words, the fluorescence signal was not involved in the assembly process but was included as a passive marker. The assembly was observed using fluorescence microscopy with an overlap of red and green signals from the two mixed populations with complementary ssDNA (see time course in Figure 4). Noncomplementary ssDNA was used in the negative control and shown as blue and green populations. The assembly timeline was studied by calculating Pearson’s coefficient from images taken at various time points (Figure 4A). The samples after mixing were placed on microscope slides at various time points to approximate the time course. The *y*-axis represents Pearson’s coefficient values, with the *x*-axis showing different time points in minutes. A Pearson’s coefficient of 0 indicates a fully disassembled state or not assembled, while a value of 1 corresponds to a fully assembled state. Assembly is typically observed as clusters of EDs (red, green, and overlapping yellow signals in the time course in Figure 4B), whereas a nonassembled state appears as a dispersed field of EDs (blue and green control in Figure 4B). Unassembled droplets are distributed over a flat surface, and sometimes the concentration of droplets is so high that they overlap spatially even though they do not assemble, making it difficult to distinguish overlapping droplets from assembled ones. This is shown as a slow but consistent rise in overlapping signals under the negative control condition in Figure 4A.

**Figure 4:**
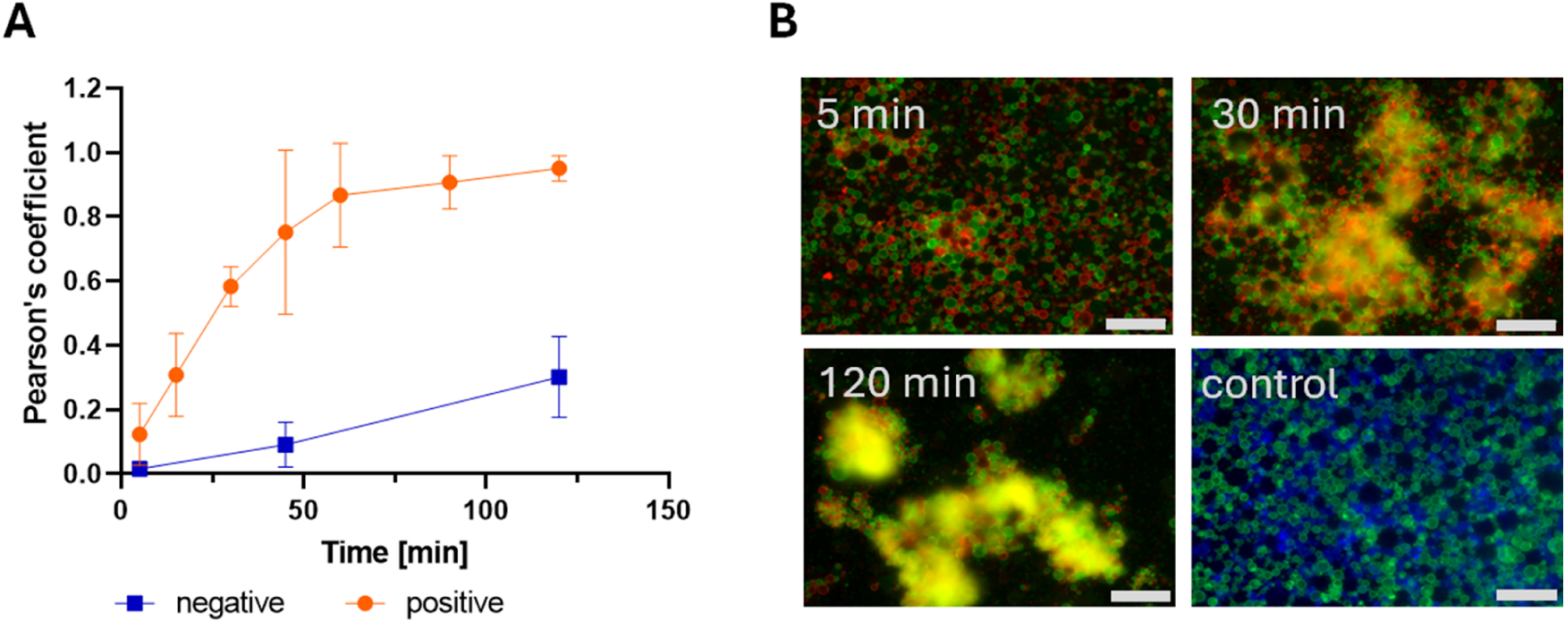
Data from the pixel-based colocalization method. (A) Time course for the assembly process using the pixel-based colocalization method, positive control (orange) and negative control (blue) using complementary and noncomplementary ssDNAs, respectively. (B) Representative fluorescence micrographs used to generate the data in panel (A). The presence of early assembled structures (complementary ssDNAs) is visible 30 min past mixing and at 2 h after assembly is complete. For reference, the micrograph of the negative control (noncomplementary ssDNAs) is also shown; Scale bar: 50 μm.

Comparing the optimal condition (1/5x B + 4x O) with the results obtained using the pixel-based colocalization method (see Figure 5), we observe notable differences in reaction dynamics. Under the pixel-based colocalization method, a plateau is reached, likely due to the compaction of droplets combined with gravitational sinking, which limits further observation of reaction progress. In contrast, with the fluorescence-based molecular beacon methods, despite the droplet compaction at the bottom of the container, particle interactions and linking continue, allowing for more comprehensive tracking of the ongoing assembly reaction.

**Figure 5:**
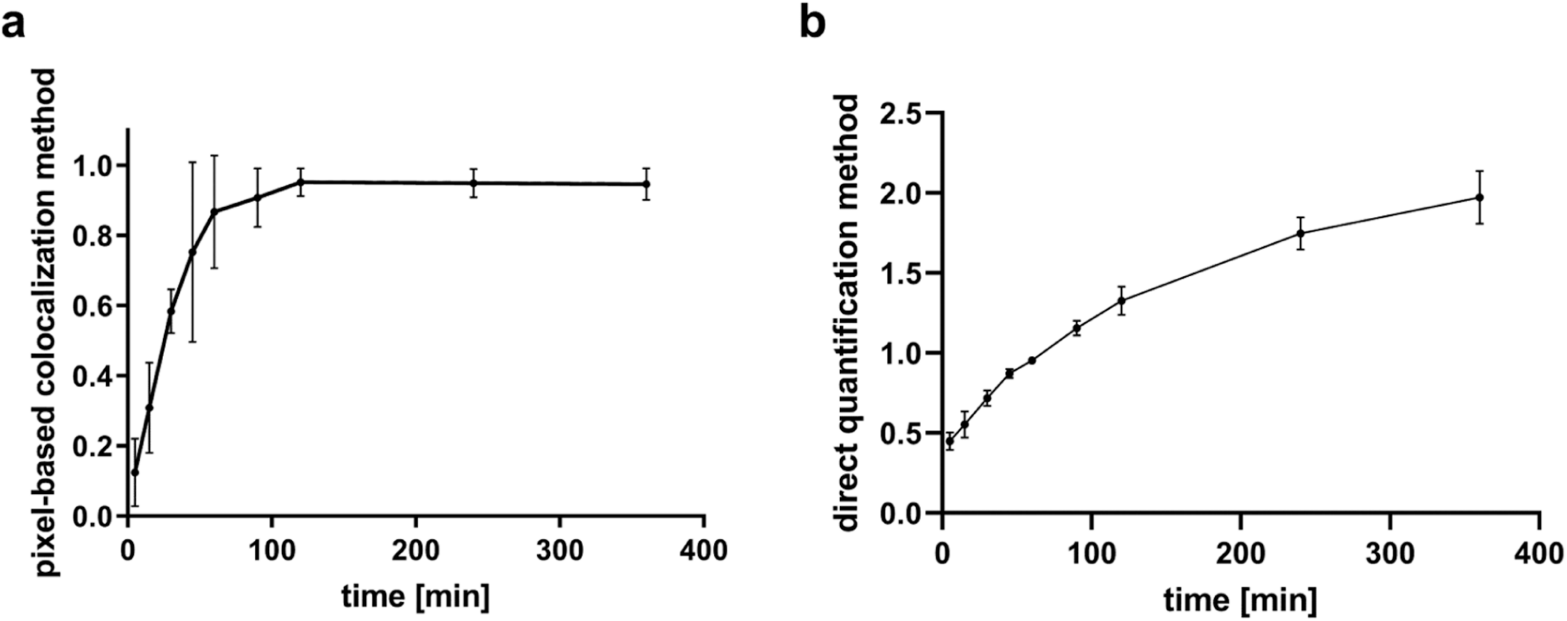
Assembly time course observed with (a) pixel-based colocalization method or with (b) beacon and direct plate reader method. Only the best ratio of beacon/opener is shown, and a comparison of methods is illustrated in Supporting Figure S5. Each point is the average of four replicates. The two models exhibit different optimal fitting profiles. The pixel-based method followed a sigmoidal trend (*R* ^2^ = 0.9948), suggestive of a cooperative or threshold-type process, while the direct fluorescence data was best approximated by a third-degree polynomial (*R* ^2^ = 0.9988), reflecting a nonlinear response.

Comparing the two methods of evaluating higher-order assembly, we note several important differences. Methodologically, the pixel-based colocalization method can accommodate a time course, but the mixed populations in one sample need to be transferred over to a thin microscope slide for analysis. We observe that this transfer step alone can disrupt higher-order assemblies and, therefore, impact the outcome of the experiment. In contrast, the fluorescence-based molecular beacon method gives real-time output from the same mixed sample without perturbation or transfer, revealing an undisturbed time course. Second, as already mentioned, we do observe an overlap of signal that is not due to assembly but purely due to proximity, dependent on the ED density. Variations in ED density could minimize such overlaps in signal but would also limit the extent of assembly in the case of complementary sequences. In cases where maximum assembly was the goal, the low-density ED populations would be counterproductive.

The fluorescence-based molecular beacon quantification and localization method not only provides qualitative insights but also delivers quantitative data. This ability to precisely measure different degrees of labeling allows for a deeper understanding as it can distinguish actual binding events and their extent. This fluorescence-based method offers real-time dynamic measurements of droplet interactions, which are essential for accurately analyzing complex assembly behaviors. This system addresses the challenge of accurately tracking molecular assembly and disassembly in real-time—key to understanding how molecules dynamically form complex structures—by providing a much-needed tool to quantify these processes.

Molecular beacons offer real-time, highly sensitive detection with sequence specificity, making them invaluable for diagnostics,^11,12^ genetic studies,^13–15^ and environmental monitoring.^16^ In synthetic biology, molecular beacons can be engineered to function as molecular switches,^17^ sensors, or regulatory elements within artificial genetic circuits. Moreover, in the realm of therapeutic applications, molecular beacons can be designed as part of smart drug delivery systems, where they could respond to specific molecular cues within a disease environment to release therapeutic agents selectively at the target site, minimizing off-target effects and improving treatment efficacy (Table 1).^18,19^

**Table 1:**
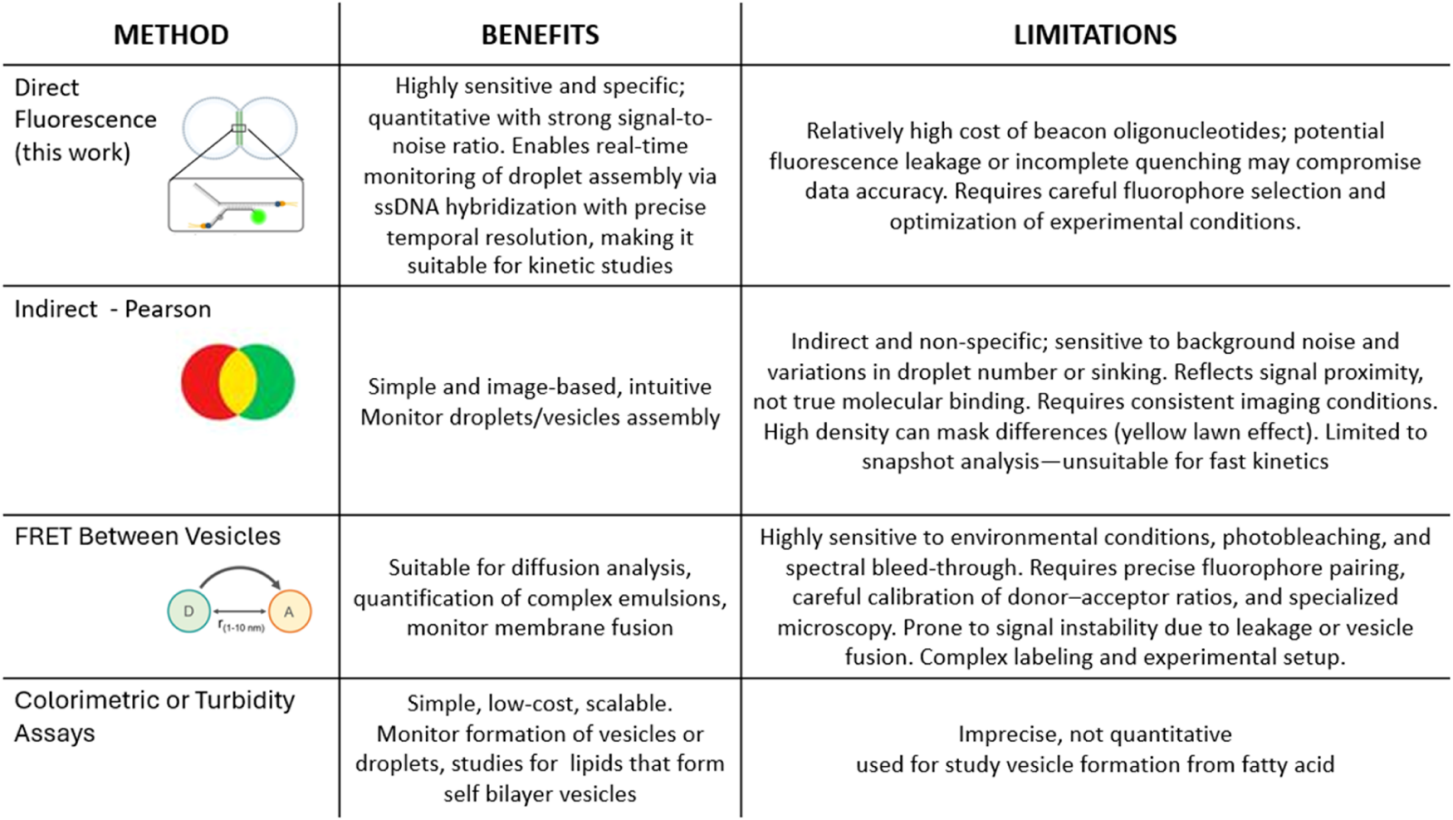
Comparison of Various Methods with Benefits, Use-Cases, and Limitations

Our fluorescence-based molecular beacon system offers distinct advantages in real-time resolution, reduced sample perturbation, and quantitative precision. While FRET has been widely used to study dynamic molecular interactions, it often requires careful donor–acceptor pair optimization, limiting its generalizability. Similarly, conventional imaging approaches may suffer from artifacts introduced during sample preparation, including a loss of spatial organization. We have shown the efficacy of using this method in vitro and on well-controlled and composed samples. The use of the beacon and opener in assessing the interactions with live cells and under more complex conditions, including blood plasma or serum, has not been tested. New experiments are being designed to be tested under such conditions. Also, the precision of the method, for example, to distinguish between single nucleotide mismatches, is still to be determined and will likely be affected by the experimental conditions. But overall, these limitations do not undermine the core utility of molecular beacons but instead underscore the need for careful system design and context-specific adaptation.

In the context of targeted drug delivery and gene therapy, precision is paramount for achieving optimal therapeutic outcomes. This technology enables more specific targeting and allows for the use of smaller, more effective dosages, thereby revolutionizing treatment strategies. The development of multifunctional, targeted drug delivery vehicles enhances the efficacy of therapeutic regimens, improving patient outcomes. Additionally, it opens possibilities in areas such as the development of smart drug delivery systems, synthetic cellular mimics, and metabolic engineering.

The strategic arrangement of droplets in a 3D configuration enables the facilitation of various biochemical reactions. This capability is particularly valuable to produce macromolecules in pharmaceutical applications where controlling the speed and quantity of reactions ensures consistency and efficiency.

Looking ahead, the fluorescence-based molecular beacon system serves as a versatile platform for exploring the idea that increased object complexity reduces the likelihood of identical copies without an information-driven mechanism. This system offers valuable insights into how specific interactions guide the assembly of complex structures. Programmable units, made possible by altering DNA sequences and modifying droplet surfaces with specific oligo sequences, offer greater versatility and control in applications ranging from genetic customization to tailored droplet behavior.^20–22^ The direct fluorescence-based molecular beacon system’s implications for understanding complexity and programmability position it as an asset for future research and technological advancements.

## Materials and Methods

The methodology is based on our previously published work^10,23^ with modifications detailed here. Beacon and opener are presented as the ratios of the pixel-based colocalization method.

### Lipid Solution Preparation

A mixture of POPC (2-oleoyl-1-palmitoyl-*sn*-glycero-3-phosphocholine, CAS 26853-31-6), DSPE-PEG2000 (1,2-distearoyl-*sn*-glycero-3-phosphoethanolamine-*N*-[methoxy­(polyethylene glycol)­2000], CAS 474922-26-4), and DSPE-PEG2000-btn (1,2-distearoyl-*sn*-glycero-3-phosphoethanolamine-*N*-[biotinyl-(polyethylene glycol)­2000], CAS 385437-57-0), all sourced from Avanti Polar Lipids (Alabaster, AL), was prepared in chloroform in a glass vial at a molar ratio of 88:11:1. Chloroform was completely removed by evaporation under vacuum until dry (room temperature, ∼10 min).

### Preparation of the Oil Phase

The lipid film was resuspended in DEP (diethyl phthalate) oil (Sigma-Aldrich, Buchs, Switzerland) to achieve a final lipid concentration of 2 mM. This lipid–oil mixture was sonicated using a Sonorex Digitec DT 156 BH sonicator (Bandelin GmbH, Berlin, Germany) for five cycles, each consisting of 10 min of sonication at 40 °C, followed by incubation at 80 °C for 10 min and a 30 s vortexing step at room temperature to ensure complete solubilization of the lipids. The resulting oil–phospholipid solution was used on the same day to ensure consistent results.

### Emulsification Process—Droplets Creation

A hosting solution (HS) containing 25 mM NaCl, 500 mM glucose, 8 mM MgCl_2_, and 50 mM HEPES at pH 7.2 in ultrapure water (all reagents were from Sigma-Aldrich, Buchs, Switzerland) was prepared to host the emulsified droplets (EDs). The HS should be stored at +4 °C and used within 1 week. Before use, allow the solution to reach room temperature.

To emulsify, 50 μL of the lipid–oil mixture was added to 450 μL of the HS in an Eppendorf tube. Emulsification was achieved by mechanically agitating the tube 35 times by drawing it back and forth on a rack (room temperature). After emulsification, the solution exhibited turbidity, indicating successful dispersion.

### Surface Functionalization of EDs and Mixture Preparations

Single-stranded DNA (ssDNA) oligonucleotides were synthesized, modified, and HPLC-purified by the supplier (Explora Biotech, Venice) and then dissolved to a final concentration of 100 μM with pure water, aliquoted in 20 μL each and stored at −20 °C. The labeling mixture was prepared with a specific volume ratio of streptavidin:biotinylated-ssDNA of 2.7:1.^10,23^ The solution was prepared and incubated 30 min at room temperature in the dark (unlabeled streptavidin, Strept.-AF350, Strept.-AF488, or Strept.-AF 647; sequences of ssDNA; see Table S1). Equal volumes of ED solution and labeling mixture were mixed with 90 min incubation with gentle agitation at 42 rpm, room temperature, in the dark. Following incubation, the decorated EDs were gently washed three times by centrifugation (3 min at 2000*g*, room temperature) to remove any unbound reagents. Fresh HS was added after each wash step and the fluorescence of the wash was measured (data not shown) to ensure complete washout from the extra label.

For the assembly process, binary mixtures of EDs, ssDNA beacon + ssDNA opener (assembly), or ssDNA beacon + ssDNA random (negative control) for the fluorescence-based molecular beacon method and binary mixtures of EDs, ssDNA1 + ssDNA3 (assembly), or ssDNA1 + ssDNA2 (negative control) for the pixel-based colocalization method were incubated at room temperature in the dark and monitored over time. Further details regarding oil and salts can be found in Supporting Information Table S1. ssDNA oligonucleotide sequences are described in Supporting Information Table S2.

### Microscopy and Image Processing

#### Pixel-Based Colocalization Method

The microscope slides were cleaned in three steps: Washed with soap and distilled water, rinsed with acetone and then ethanol, and dried with nitrogen gas. The imaging chamber was prepared using a press-to-seal silicone isolator (CultureWell Press-To-Seal Silicone Isolator-CWS-13R-0.5, 0.5 mm). Binary mixtures were prepared as follows: Assembly: ED-ssDNA1 + ED-ssDNA3; Negative Control: ED-ssDNA1 + ED-ssDNA2. The mixtures were incubated for up to 2 h at room temperature and then transferred to the microscopy chamber without resuspension and sampled at specific intervals (5, 15, 30, 45, 60, 90, and 120 min). The samples were evaluated using an inverted light and fluorescence microscope Fluorescence X-CITE 120Q light source and using a 20× objective, AxioCam MRm Monochrome camera. Images were captured with the following filter settings for the excitation: 470:40 nm (green), 545:25 nm (red); for the beam splitter: 495 nm (green), 570 nm (red); and the emission: 525:50 nm (green), 605:70 nm (red). A custom macro for ImageJ, developed by the Advanced Imaging Facility at CIBIO, University of Trento, was used to analyze specific images for Pearson’s coefficient. The Pearson correlation coefficient measures the linear correlation between the two variables, quantifying the strength of their relationship. This coefficient ranges from −1 to +1, with +1 indicating a perfect positive correlation, 0 indicating no correlation, and −1 indicating a perfect negative correlation.^24^ Images were contrast-adjusted, and channels were overlaid using ImageJ (version 1.53q). Graphs were Created in https://BioRensder.com.

#### Direct Fluorescence-Based Molecular Beacon Method

Binary mixtures were prepared as follows: Assembly: ED-ssDNABeacon + ED-ssDNAOpener; Negative Control: ED-ssDNABeacon + ED-ssDNARandom. Autofluorescence controls: Unlabeled ED, ED-ssDNABeacon, Hosting solution. Each mixture was equally distributed in a 384-well Black Greiner Bio-One μClear plate clear bottom in quadruplets. Fluorescence was captured by a Varioskan LUX, software version: SkanIt Software 6.0.2 for Microplate Readers RE, ver. 6.0.2.3; 384-well adapter for plate with lid; total time reading 12 h, interval steps 5 min, excitation 490/12 nm; emission 525 nm, optics top; temperature ranging 20/24 °C. Total reading duration: 12 h.

## Supplementary Material





## Notes

Melting curve (Figure S1); kinetics of beacon–opener interaction (Figure S2); automatic microscope micrograph of assembly with beacon-opener (Figure S3); stability and autofluorescence controls of the beacon system (Figure S4); and cartoon of droplets assembly of ssDNA1 and ssDNA3 vs ssDNA beacon and ssDNA opener (Figure S5) (PDF)
List of chemicals, CAS number, and vendor (Table S1); ssDNA oligo list, modification, and length (Table S2) (PDF)
