# Supplementary material for: Real-Time Fluorescence-Based Method for Dynamic Quantification of Droplet Network Assembly
Source: ACS Omega. 2025 Jun 2;10(22):23528–34. doi: 10.1021/acsomega.5c02156 (PMC12163661; doi:10.1021/acsomega.5c02156)
Supplement: Supplementary file 1 [file ao5c02156_si_001.pdf]

Real-time fluorescence-based method for dynamic quantification of droplet network assembly

AUTHORS: Alessia Faggian 1, Federica Casiraghi 1, Martin M. Hanczyc1,2 *

1 Laboratory for Artificial Biology, Department of Cellular, Computational and Integrative Biology, University of Trento, Via Sommarive, 9 - 38123 Povo, Italy

2 Chemical and Biological Engineering, University of New Mexico, Albuquerque, NM 87106

****Corresponding author***

SUPPLEMENTARY INFORMATION

Characterization of the Molecular Beacon.

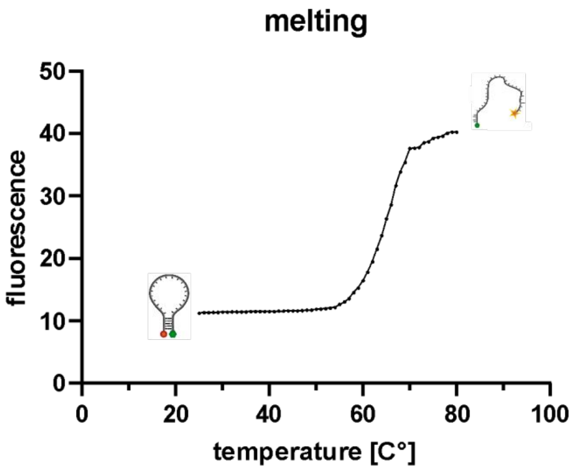

Figure. S1. Melting curve. The fluorescence intensity of the solution containing the molecular beacon is plotted as a function of temperature on a linear scale from 25 to 80 in 1 °C increments. The increase of fluorescence seen with the increasing of temperature is due to the change in the structure of the sequences from a steam-and-loop structure in which the fluorophore is quenched to a random coil where the fluorophore is unquenched. Fluorescence levels were monitored during each hold, and this process was repeated for a total of five loops of measurement.

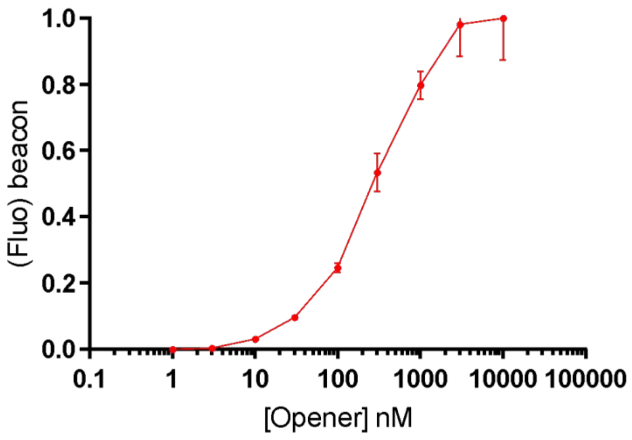

Figure S2: Binding curve for opener–beacon hybridization to saturation. (data are normalized) The curve depicts the fluorescence intensity of the beacon as a function of increasing concentrations of the opener.

binding curve method: Melt (till 80°C) and cool down beacon to ensure all starts from close. Series of reaction mixtures in which the concentration of the beacon is kept constant (30 nM); Vary the concentration of the opener sequence across a wide range (e.g., from 0.1 nM to 100 µM, logarithmically) to observe the hybridization kinetic response. Fluorescence has been measured with a plate reader in a 384 wells plate (black walls, glass bottom - 384 well, sensoPlate, Greiner bio-one).

Microscopy

In a 384 well plate (black walls, glass bottom - 384 well, sensoPlate, Greiner bio-one) 40 µl of total volume (20µl + 20µl of binary mixture) has been distributed. The solution contains droplets labeled with 100% beacon, 100% opener, or a mix of both. Instrument Image Xpress® Micro Confocal: total time reading 16 h, intervals step 10 min; excitation Cyan: 475/28 nm; emission filter 536/40 nm, dichroic filter 506 nm, mosaic.

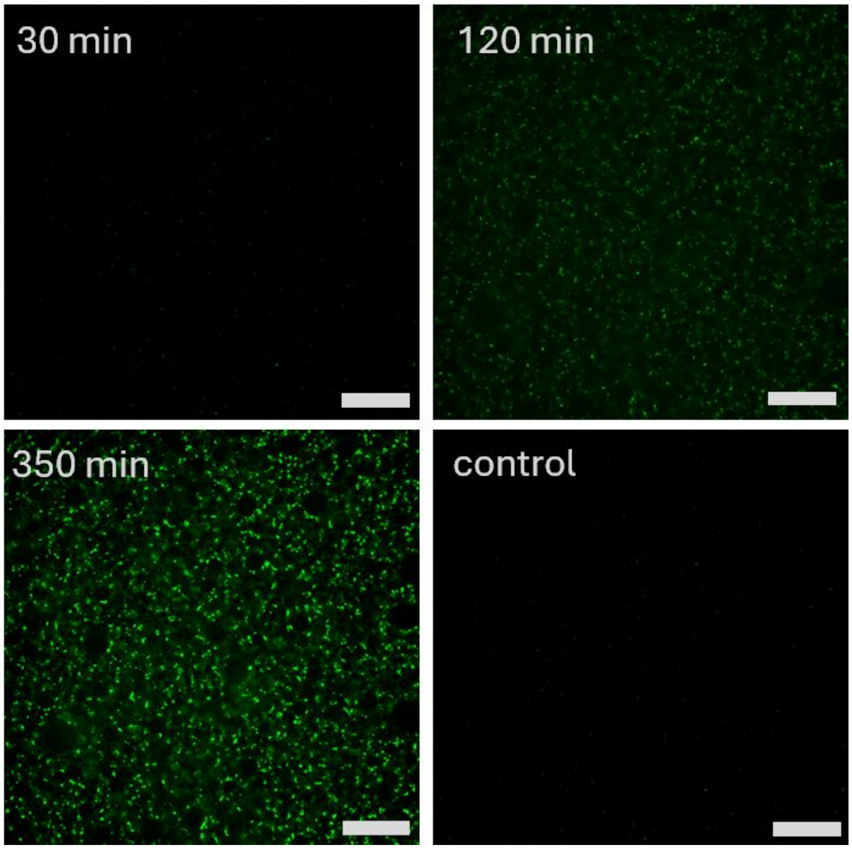

Figure S3. Population-level assessment of beacon activity. Time course for the assembly process using the molecular beacon-based method. The fluorescent signal for the binding of the DNA beacon and its opener starts to be visible after 120 min. Negative control is included for reference. Scale bar: 150 *μm.*

Stability and Autofluorescence Controls

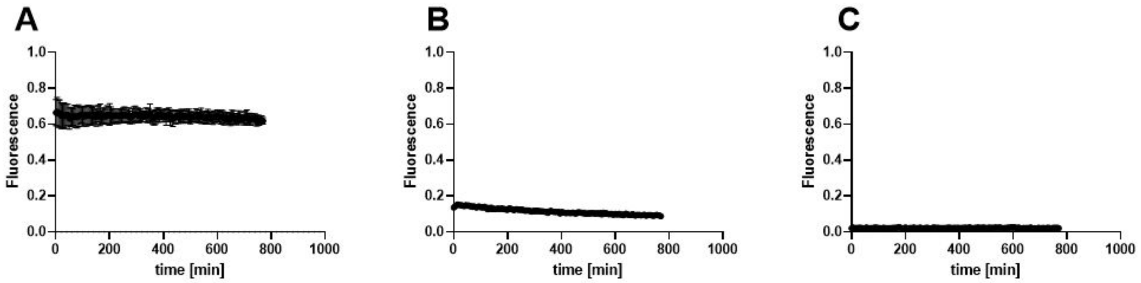

Figure S4: Fluorescence readings of the beacon system. A) The resulting assembly of droplets labeled with the beacon and droplets labeled with the opener (Figure S5 B) was monitored for an additional 800 minutes to assess structural stability and potential photobleaching. The fluorescence signal remained stable over time, with no detectable photobleaching. B) Beacon-labeled droplets alone exhibited very low autofluorescence and C) Opener-labeled droplets alone showed no detectable autofluorescence.

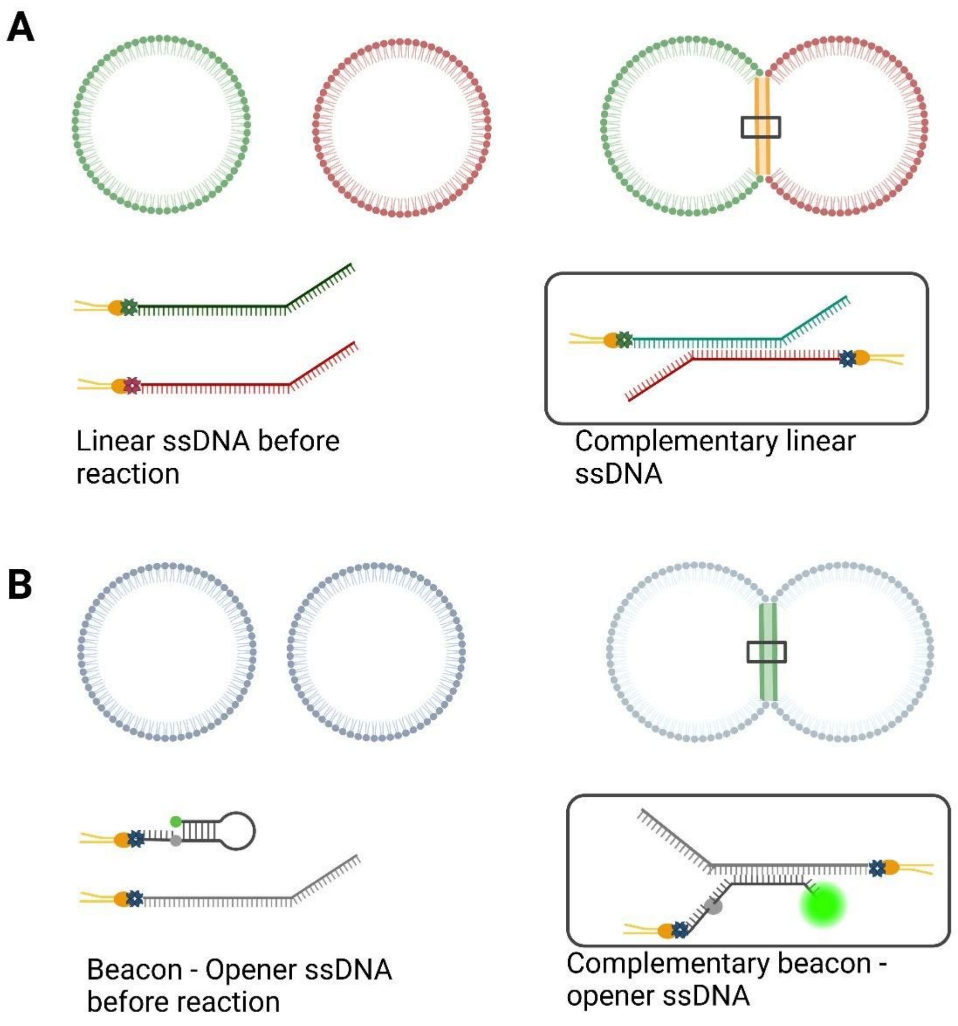

Figure S5:

A) Pixel-based colocalization method.

B) Direct fluorescence-based molecular beacon method.
