# Supplementary material for: Real-Time Fluorescence-Based Method for Dynamic Quantification of Droplet Network Assembly
Source: ACS Omega. 2025 Jun 2;10(22):23528–34. doi: 10.1021/acsomega.5c02156 (PMC12163661; doi:10.1021/acsomega.5c02156)
Supplement: Supplementary file 2 [file ao5c02156_si_002.pdf]

Real-time fluorescence-based method for dynamic quantification of droplet network assembly

AUTHORS: Alessia Faggian 1, Federica Casiraghi 1, Martin M. Hanczyc1,2 *

1 Laboratory for Artificial Biology, Department of Cellular, Computational and Integrative Biology, University of Trento, Via Sommarive, 9 - 38123 Povo, Italy

2 Chemical and Biological Engineering, University of New Mexico, Albuquerque, NM 87106

****Corresponding author***

SUPPLEMENTARY INFORMATION

MATERIALS		CAS	Company
POPC	2-oleoyl-1-palmitoyl-sn-glycero-3-phosphocholine	26853-31-6	AvantiLipids
DSPE-PEG2000	1,2-distearoyl-sn-glycero-3-phosphoethanolamine-N-[methoxy(polyethylene glycol)2000]	474922-26-4	AvantiLipids
DSPE-PEG2000-btn	1,2-distearoyl-sn-glycero-3-phosphoethanolamine-N-[biotinyl-(polyethylene glycol)2000]	385437-57-0	AvantiLipids
DEP oil	Diethyl Phthalate	84-66-2	Sigma-Aldrich, Buchs, Switzerland
Chloroform		67-66-3	Sigma-Aldrich, Buchs, Switzerland
Glucose		50-99-7	Sigma-Aldrich, Buchs, Switzerland
HEPES	4-(2-hydroxyethyl)-1-piperazineethanesulfonic acid	7365-45-9	Sigma-Aldrich, Buchs, Switzerland
NaCl	sodium chloride	7647-14-5	Sigma-Aldrich, Buchs, Switzerland
MgCl2	magnesium chloride	7786-30-	Sigma-Aldrich, Buchs, Switzerland

Table 1. List of Materials to prepare the droplets.

NAME	SEQUENCE	BP	5'-Mod.	Internal mod.	3'-Mod.
ssDNA #1	AAAGATTACACACGA	15	Biotin	None	None
ssDNA #2 neg	CAT CCA TGG TGG AGG	15	Biotin	None	None
ssDNA #3	TCGTGTGTAATCTTT	15	Biotin	None	None
ssDNA beacon	AAAAAAA[A]CCATGCGCACCCAGTTGTGTTGCAGAT TCGCATGGT	44	Biotin	BHQ2	Cyanine3
ssDNA opener	AAAATTGCTGTCTAATGATAAATCTGCAACACAACTG GGTGCATGGT	47	Biotin	None	None
ssDNA random	CCGTGTACCTACCAAACCTCTTTAATCTAAGTTCAGA CTAATTGGTA	43	Biotin	None	None

Table 2. single strand DNA (ssDNA) oligo sequences, length and modifications for each labeling.
